# Bridging research and clinical care – the comprehensive cancer centre

**DOI:** 10.1002/1878-0261.12442

**Published:** 2019-01-23

**Authors:** Simon Oberst

**Affiliations:** ^1^ Cancer Research UK Cambridge Centre UK

**Keywords:** accreditation, comprehensive cancer centre, outcomes, quality

## Abstract

Comprehensive cancer centres (CCCs) are at the heart of the landscape of cancer research, education and care in Europe. They are vital hubs where the historic gaps in the research to clinical care continuum are bridged. CCCs have established hallmarks, but a greater emphasis is needed in Europe to create more effective CCCs using the partnership model of university medical centres and university research departments and institutes. This review will summarise the organisational structures and processes essential for producing quality outcomes for patients and effectiveness in the translational process. The Organisation of European Cancer Institutes and European Academy of Cancer Sciences have established complementary quality accreditations systems to test the clinical and research excellence of CCCs. The EU should have an ambition to create more CCCs based on university hospitals, for each 5–10 million population and in every Member State.

**Abbreviations**
CCC: comprehensive cancer centre
EACS: European Academy of Cancer Sciences
NCI: National Cancer Institute
OECI: Organisation of European Cancer Institutes

## 1 Introduction

If we think back far enough, the original idea of a comprehensive cancer centre (CCC) was promulgated by William Marsden in 1851 in London. Deeply affected by the death of his wife from cancer, Marsden resolved to classify tumours, research the causes and find new treatments for his patients at ‘The Cancer Hospital (Free)’. The formal Institute, which became the Institute for Cancer Research, was founded in 1909. In Paris in 1918, Marie Curie and Claudius Regaud proposed a general development project for the Institut du Radium, where research and therapeutic applications would be closely linked. Thus, the well‐known research to care continuum was born, and remains the main concept of CCCs.

Decades after Marsden and Curie, the need for consistency and safety in administering systemic therapies was the catalyst for bringing all cancer services and clinical trials together in multimodality centres, moving on to accrue expertise in translational research. As noted elsewhere in this volume, the Eurocan+Plus project also stressed the importance of CCCs as structures that provide multidisciplinary academic expertise covering a substantial spectrum linking research and health care. In the United States, CCCs have been formally recognised for a long time by the National Cancer Institute (NCI). In the United Kingdom, the groundbreaking Calman–Hine report in 1995 emphasised the need for formalised collaboration both within cancer centres and between all healthcare providers, and gave rise to the cancer networks in England (Calman and Hine, 1995).

In Europe, CCCs have developed in various forms, largely influenced by the health systems of Member States of the EU, and those forms cohere around two basic types:

1. The most obvious form of CCC is the standalone specialist cancer centre, examples of which include the Netherlands Cancer Institute (NKI‐AVL) and the Royal Marsden Hospital/Institute of Cancer Research in the United Kingdom. These centres often have a long history of specialist cancer treatment and translational research and have links with Universities.
2. The second major form of CCC apparent in Europe is that which is a partnership of a university, a university hospital and associated institutes working in the cancer field. Examples of this form include the Cancer Research UK Cambridge Centre and the Oslo Comprehensive Cancer Centre. Often, these types of CCC have been formally brought into being only in the last 5–15 years as an answer to the critical challenge to integrate translational research and high‐quality care in an academic environment.

## 2 Hallmarks of CCCs

The hallmarks of a CCC with either of these forms have been recognised as being:

- Excellence in diagnosis, treatment and care of patients based on multidisciplinary team working
- High‐quality outpatient and inpatient facilities delivering an optimal patient experience
- A strong clinical trials infrastructure and a breadth of open clinical trials with chief/principal investigators drawn from the CCC staff, and a high rate of accrual of new cancer patients
- High‐quality diagnostics, and capabilities in molecular pathology and molecular imaging as well as histopathology
- Translational science with breadth and depth, bringing preclinical science to clinical implementation
- A consistent academic output in highly rated journals across a wide spectrum of disciplines
- Evidence of innovation in patents, spin‐off companies, and practice changes mediated through national bodies and regulators
- Excellent e‐hospital and information systems which allow the collection of clinical data and linking these with Big data analytics for research
- Educational programmes which comprehensively cover education and training of cancer clinicians and scientists, and the education and support of patients and their carers
- Good career advancement opportunities for staff
- A commitment to networking across the population served, linking (with ICT interoperability infrastructure) to other hospitals, primary care, and supportive and palliative care services
- Integration with national prevention, screening and early detection strategies
- Many CCCs also have incorporated, or have access to, high‐quality basic science in cancer, embracing the biological and physical sciences, as well as mathematics.

These hallmarks are the domains which form the core of the Accreditation and Designation Programme (http://www.oeci.eu/Accreditation/Page.aspx?name=BACKGROUND) of the Organisation of European Cancer Institutes (OECI), which is the only pan‐European accreditation programme for CCCs and clinical cancer centres. Launched in 2008, the programme was a major response to the need for an independent mechanism for assessing and certifying quality in comprehensive cancer care, and as a tool to help centres improve in their own settings. ‘It's not enough for a cancer hospital simply to say they are one of the top centres – they need to show they are’ (http://cancerworld.net/cover-story/mahasti-saghatchian-pioneering-a-quality-mark-for-europes-cancer-centres/). By 2020, the OECI programme will have accredited 50 large centres in Europe, of which approximately half will be designated as CCCs. The unique feature of this programme – the only cancer accreditation programme to be accredited by the International Society of Quality in Healthcare – is that it examines both clinical care delivery, and the flow of clinical and translational research into the clinic, using qualitative standards and quantitative metrics. The emphasis is on a quality system across all tumour types which monitors and informs improvements in diagnostics, all treatment modalities, nursing, survivorship and end‐of‐life care. In Germany, German Cancer Aid accredits and designates a dozen research‐strong CCCs (https://www.krebshilfe.de/informieren/ueber-uns/deutsche-krebshilfe/about-us-deutsche-krebshilfegerman-cancer-aid/), whilst the German Cancer Society accredits their clinical oncology services per tumour type.

## 3 Organisational structures of CCCs which catalyse high quality and outcomes

The ability of these CCCs to bring these components together in a continuum which delivers integrated research, outstanding innovation and excellent patient outcomes requires a clear organisational structure, sustainable resources and excellent leadership. Since this is more of a challenge for the CCCs based on a partnership model between a university and a university hospital rather than one legal entity, it is appropriate to examine how this can work optimally in that model.

Writing on matrix management in the Harvard Business Review, Bartlett and Ghoshal observe that creating a collaborative mentality in leaders from different institutions is a greater challenge than creating the matrix structure itself (Barlett and Ghoshal, 1990). Fundamentally, under a controlling board, the leadership of the CCC needs to be configured in such a way as to bring scientists from various university departments and institutes, and clinicians, into collaboration in programmes which are clearly defined and governed. These could be disease‐specific programmes such as for breast or gastrointestinal cancers, or cross‐cutting programmes such as imaging or early detection. The aim here is to break down the silo culture and foster collaborations, leading to clinical research and practice changes which would otherwise remain dormant or be delayed.

What can give these programmes extra potency is to provide each with an infrastructure budget (above and beyond the usual competitive grant funding or institute core funding arrangements) for which each programme is then accountable to both the overall CCC Director and also the examination of peers. The power of face‐to‐face contact between people of different disciplines, and the incentive of budgets to accelerate programmes, should never be underestimated. In the United Kingdom, the funding of 14 translational centres (and 18 Experimental Cancer Medicine Centres, https://www.ecmcnetwork.org.uk/) by Cancer Research UK (https://www.cancerresearchuk.org/funding-for-researchers/our-research-infrastructure/our-centres) and the National Institute for Health Research has been fundamental to achieving the development of collaborative infrastructures in translational research, and CRUK's designation of ‘Major Centres’ (at the time of writing just at Cambridge and Manchester) brings more funding for cross‐collaboration, pump‐priming of innovations and training the next generation of clinician scientists.

In a matrix organisation, there is a constant need for the CCC leadership to be shuttling between the university hospital and the university departments and institutes, establishing coherent research and clinical strategies for the CCC, underpinned by the funding of necessary infrastructure (for instance, sequencing, molecular diagnostics and radiology). The experience of 10 years of the OECI Accreditation and Designation Programme has enabled the organisation to advise centres on how to strengthen organisational structures and accountabilities, particularly in a university hospital context. Examining governance structures and strategies for care delivery and research is one of the key components of the assessment.

## 4 Quality at team levels and quality at the centre level are complementary

Multidisciplinary teams in the hospital have long been recognised as vital tools for high‐quality patient diagnosis and treatment (Brar *et al*., 2014) within the broader continuum of the whole patient pathway ‘from home to home’. But it is less well observed how important the role of clinician scientists, clinical fellows, principal investigators and research nurses is within MDTs, to follow each case coming for discussion, and consider the appropriateness for a clinical trial. In a well‐functioning CCC, the majority of patients of reasonable fitness could be considered as candidates for a suitable clinical trial (Bouvier *et al*., 2007). Furthermore, in the genomic age every patient should be asked for consent for samples for tracking and monitoring disease response or progression and for future research. Indeed, in leading CCCs, for certain cancers, every patient is being offered longitudinal deep genome and RNA sequencing for clinical and research purposes (De Mattos‐Arruda and Caldas, 2016), and this is currently expanded to include immune profiling and proteomics in a wide ‘precision medicine’ paradigm. This comprehensive level of endeavour – supported by Molecular Tumour Boards – is optimally resourced in CCCs.

In the United States, the Lancet Oncology Commission report (Jaffee *et al*., 2017), building off the Moonshot programme, details the complexity of research, data‐sharing and public health effort required to achieve these step changes. A huge and sustainable investment in the infrastructure of molecular analysis is required, together with a responsive and educated clinical interface, to ensure that these insights are actionable and that tumour responses are monitored and fed back into the data lakes. CCCs are undoubtedly at the heart of this co‐ordinated programme.

The importance of the CCC structure is complementary to the insights of Michael Porter and teams at the Harvard Business School in the development of Integrated Practice Units (https://www.isc.hbs.edu/health-care/vbhcd/Pages/integrated-practice-units.aspx). Evidence in the United States and Germany suggest how these IPUs improve clinical effectiveness and cut costs by the team taking responsibility for the whole patient pathway and by monitoring detailed data points and patient outcomes with great care. Nevertheless, partly because of molecular insights shared between tumours sites (Robinson *et al*., 2017), and partly through the value of learning from other teams, the colocation of IPUs within a CCC leverages more power in translational research.

## 5 Clinical evaluation and real‐world data

The benefits of colocation and integrated working in CCCs are most keenly experienced in bridging the common translational research gaps identified (Celis and Pavalkis, 2017). The first of these is between preclinical research and clinical research – establishing early clinical trials with novel and adaptive designs which leverage molecular technologies, complex bioinformatics, biostatistics and pharmacology. CCCs are the powerhouses of providing chief investigators and key cross‐disciplinary collaborators for such trials and the infrastructure to support them. The second gap relates to establishing late translational research, clinical validation and assessment of clinical effectiveness. Here again, the CCCs excel in having access to significant cohorts of patients, a high accrual rate of patients to trials (often 15–40%), and a ready access to national evidence review groups to establish fast implementation at a national scale.

These CCC advantages are underpinned by a wealth of real‐world data of their patients, through electronic health records linked to multiple data from laboratory analyses. Generally, the CCCs are able to follow their cohorts of patients longitudinally, and therefore track disease response, resistance and late effects of treatment for the patients over many years, thereby building up powerful data lakes/warehouses for research and development (Foran *et al*., 2017). Interaction with population‐based registries, and tracking the long‐term outcomes of treatment, will be vital developments in the coming years. These data warehouses will be fruitful platforms for research around survivorship, which takes seriously the reality of cancer as a long‐term condition, and validation of quality‐of‐life indicators, which are crucial to supporting patients living beyond cancer. This area of research has been underinvested in the past, especially in the area of prevention or early detection of recurrence, but there is evidence in some OECI‐accredited CCCs that research programmes in survivorship are using cohort studies for predictive prevention. The data generated will also be essential for further studies in the health economics of cancer.

These opportunities and challenges around translational research and the need to stimulate innovation in CCCs are what has led the European Academy of Cancer Sciences (EACS) to develop the assessment methodology of the designation of research excellence, which assesses the research environments, strategy and outputs of CCCs in great depth, and will lead to heightened collaborations in cancer science (Rajan *et al*., 2016).

## 6 Patient outcomes in CCCs

Behind all the discussions about the ‘Added Value’ of CCCs in terms of resources and processes, there lie the ultimate tests of patient outcomes, and until recently, there have been relatively few data on the differentiation of CCCs. However, there is emerging evidence in the United States that patients treated in CCCs have better survival outcomes than those treated in other hospitals and other institutions. In a key analysis, Pfister *et al*. (2015) demonstrate on the basis of Medicare risk‐adjusted cohorts that cancer patients treated in the 11 largest freestanding CCCs in the United States have a risk‐adjusted probability of death 10% lower than among cancer patients treated at U.S. community hospitals. Patients treated at the remaining 32 NCI‐accredited CCCs and at academic teaching hospitals had average 1‐year outcomes lying between those two groupings. These differences are greater than could be explained by any change in mix based on stage of diagnosis. Since it is a policy imperative in Europe that inequalities of care should be reduced, these indications are a major challenge to the thinking around organisation of care and integration of research.

## 7 CCCs in relation to cancer care at the population level

This chapter has described the role of CCCs in the landscape of cancer care, education and research. However, the CCCs are only a part of the cancer care and research infrastructure in Europe, most patients (estimated at 80–90%) being treated outside CCCs. The role of CCCs as referral centres, and hubs of networks in the wider geography, is vital to ensure the spread of innovation and equity of access to care, as will be expanded upon in more detail in another chapter of this issue.

## 8 Future directions

Given the strengths and functions of CCCs as outlined above, what should be the direction of public policy in the EU and Member States regarding CCCs? There is a legitimate and growing ambition that there should be a CCC in every EU Member State and for every 5–10 million population. This will surely not come about by creating brand new standalone CCCs, but rather by re‐organising many university medical centres and university research departments to form stronger CCCs with the organisational structures and strengths outlined in this chapter. This will require resource, external advisory work and accreditation, but it is a realistic ambition, and one to which organisations such as OECI and EACS are keen to contribute.

## 9 Conclusions

As we move forward in the genomic age, the role of CCCs (working together in national and international networks of collaboration) is certain to become even more crucial in achieving step changes in cancer prevention, diagnosis and treatment. The colocation of multidisciplinary teams with each other, and with pathologists, radiologists, cardiologists, geneticists, molecular biologists, study nurses, together with engineers, physicists, mathematicians, computer scientists and bioinformaticians, is going to be fundamental to transformative developments in cancer research and practice changes. This is the foundational purpose of CCCs, and justifies all the current initiatives to provide accreditation programmes to enable CCCs to enhance their work, and to network effectively. It also acts as a stimulus to the ambition that there should be a CCC in every EU Member State and for every 5–10 million population.

## Conflict of interest

The authors declare no conflict of interest.
